# ICOS^+^ Tregs: A Functional Subset of Tregs in Immune Diseases

**DOI:** 10.3389/fimmu.2020.02104

**Published:** 2020-08-28

**Authors:** Dan-Yang Li, Xian-Zhi Xiong

**Affiliations:** Department of Respiratory and Critical Care Medicine, NHC Key Laboratory of Pulmonary Diseases, Union Hospital, Tongji Medical College, Huazhong University of Science and Technology, Wuhan, China

**Keywords:** ICOS, Treg cells, autoimmune disease, neoplasm, immunotherapy

## Abstract

Recent studies have reported the pathological effect of ICOS^+^ T cells, but ICOS signals also widely participate in anti-inflammatory responses, particularly ICOS^+^ regulatory T (Treg) cells. The ICOS signaling pathway endows Tregs with increased generation, proliferation, and survival abilities. Furthermore, there is enough evidence to suggest a superior capacity of ICOS^+^ Tregs, which is partly attributable to IL-10 induced by ICOS, yet the associated mechanism needs further investigation. In this review, we discuss the complicated role of ICOS^+^ Tregs in several classical autoimmune diseases, allergic diseases, and cancers and investigate the related therapeutic applications in these diseases. Moreover, we identify ICOS as a potential biomarker for disease treatment and prognostic prediction. In addition, we believe that anti-ICOS/ICOSL monoclonal antibodies exhibit excellent clinical application potential. A thorough understanding of the effect of ICOS^+^ Tregs and the holistic role of ICOS toward the immune system will help to improve the therapeutic schedule of diseases.

## Introduction

Inducible T-cell costimulator (ICOS) is a homodimeric protein with a molecular weight of approximately 55∼60 kD that was originally discovered on the surface of T cells upon T-cell receptor (TCR) stimulation in 1999 (1). As the third member of the CD28 super family, the structure and function of ICOS have many similarities with CD28 despite some differences, with the major difference being that ICOS cannot be constitutively expressed on resting T cells. Exhibiting no binding with B7-1/B7-2, ICOS has a unique ligand, ICOSL, which is expressed on the surface of many antigen presenting cells (APCs), such as B cells, dendritic cells (DCs), macrophages, and other cell types from non-lymphoid tissue, including fibroblasts, endothelial cells and epithelial cells (2). Intriguingly, ICOSL has also been detected on a small subset of T cells, accounting for 5% of CD3^+^ T cells, although the details regarding this population remain unknown (3). Recently, many researchers have investigated the distribution of ICOS on T cells, and the expression of ICOS on Th1, Th2, Th17, T follicular helper (Tfh) cells, T follicular regulatory (Tfr) cells, Tregs, type 1 regulatory T (Tr1) cells, and innate lymphoid cells (ILCs) have been successively reported, showing its indispensable role in immune responses (4–6). Taking into consideration the complexity of ICOS distribution on T cell subsets and its diverse effect toward each subset, the role of ICOS in various diseases can hardly be summarized in one word. The overexpression of ICOS could either lead to Th1 or Th2 dependent immune responses, among which regulatory T cells are an important counterbalance in the inflammatory state (7).

First in mice (8) and then in humans (9), researchers discovered a correlation between the highest level of ICOS on CD4^+^ T cells with the generation of the anti-inflammatory cytokine IL-10, demonstrating the central role of ICOS in the differentiation and function of FOXP3^+^ Tregs. Subsequently, numerous studies have been published to elucidate the role of ICOS^+^ Tregs in homeostasis and different disease conditions. Herein, we describe the phenotype and function of ICOS^+^ Tregs and summarize ICOS-associated signaling pathways of Tregs and their relationship with two important cytokines, IL-2 and IL-10. We also compare ICOS signal transduction with CD28- and CTLA-4-mediated signaling pathways, and describe their synergetic effect on anti-inflammation. In addition, we highlight the crucial role of ICOS^+^ Tregs in various immune diseases and outline its diagnostic and therapeutic effect in autoimmune responses.

## ICOS and Tregs

### Origin and Phenotype of ICOS^+^ Tregs

In homeostasis, ICOS^+^ T cells only account for a small subset of CD4^+^ T cells in peripheral blood, while they are particularly enriched in FOXP3^+^ Tregs, accounting for approximately 20% of Tregs, with most ICOS^+^ Tregs being CXCR3^+^ or CXCR3^–^CCR6^+^CCR10^–^, defined as Th1-like or Th17-like Tregs, respectively (10). The much higher proportion of CD4^+^ICOS^+^ Tregs among CD4^+^ Tregs than that of CD4^+^ICOS^+^ T cells among CD4^+^ T cells begs a question: where do these ICOS^+^ Tregs come from? During the development of thymocytes, only T cells with high-affinity TCR evolve into Tregs, and combined with the consideration that the recognition of antigens with TCR is a prerequisite for the induced expression of ICOS, it was supposed that the increased proportion of ICOS in Tregs was partly induced by these self-antigens presented by medullary thymic epithelial cells (mTECs). This opinion has been put forward by Ito et al. (2008), who observed that both ICOS^+^ and ICOS^–^ Tregs existed in both newborn thymus and cord blood, and 50% of the ICOS^+^ Tregs from cord blood expressed CD31, suggesting it contains recent thymic emigrants (9). In addition, mTECs can induce IL-2 overproduction by CD4^+^CD25^–^ T cells via ICOSL to promote Treg proliferation, providing additional evidence for the existence of ICOS^+^ Tregs in the thymus (11). However, almost all of these ICOS^+^ Tregs in adult blood were CD45RO^+^, contrasting with the phenotype of ICOS^–^ Tregs, which still included some CD45RA^+^ Tregs, and ICOS^+^ Tregs in cord blood, which expressed high levels of CD45RA (9). This phenomenon suggested that most of the ICOS^+^ Tregs in adult blood are actually effector/memory Treg cells. Similarly, although ICOS is expressed in small amounts in CD44^lo^CD62L^hi^ cTregs, ICOS is highly expressed in murine splenic CD44^hi^CD62L^lo^ eTregs (12). Most of these eTregs are Helios-positive, indicating they are mostly derived from the thymus (12). Moreover, Nicolas and colleagues have observed the expansion of ICOS^+^ Tregs from the pool of naturally occurring Tregs (nTregs) after 2,4-dinitrofluorobenzene (DNFB) sensitization in mice and ICOS^+^ Tregs being generated from ICOS^–^ precursors (13). In addition, using T cell adoptive transfer experiments, they did not observe a generation of adaptive Tregs (aTregs) with ICOS expression from FOXP3^–^ T cells after DNFB sensitization (13). However, by eliminating thymus-derived Treg (tTreg, also known as nTreg) development using Rag1^–/–^ mice, Ashley et al. have demonstrated that ICOS deficiency can reduce the accumulation of peripherally induced Tregs (pTregs, also known as aTregs) in the large intestine, indicating an important role of ICOS for the generation of pTregs under steady state conditions (14). Therefore, ICOS^+^ Tregs can be generated from both the thymus and peripheral tissues, which can be expanded rapidly or transformed from ICOS^–^ nTregs after antigen stimulation. Additionally, we suspect that the higher ICOS expression levels in Tregs than in other CD4^+^ T cell subsets could be beneficial for the maintenance of immune homeostasis, as the superior suppressive ability of ICOS^+^ Tregs could make them more efficient and capable of preventing the human body from producing overactive inflammatory responses to daily low-dose harmless antigenic stimulation.

Recently, various studies have investigated the phenotype of ICOS^+^ Tregs in diverse diseases or in the steady state. In general, Tregs with ICOS expression also coexpress many immunosuppressive receptors, such as CTLA-4, PD-1, TIGIT, and Lag3, and exhibit a higher secretion of IL-10, providing them with superior suppressive potential (13, 15). However, it is not clear if ICOS expression or signaling accounts for these observations. The co-expression of these molecules may be a reflection of the fact that they are both highly expressed by eTregs. ICOS, as a co-stimulatory molecule, does not have a direct inhibitory function *per se*. Whether the ICOS signaling could influence the expression of other co-inhibitory molecules needs further exploration, and some of the relationships between ICOS and CTLA-4 or IL-10 are discussed below (in section “ICOS and CTLA-4” and section “The Role of ICOS Signaling in Tregs,” respectively).

Furthermore, ICOS^+^ Tregs also upregulate many genes associated with TCR stimulation and appear to be more proliferative. Some transcription factors that are usually associated with T helper cell differentiation have been observed to participate in the biology of eTregs and to be associate with ICOS expression. For example, B lymphocyte induced maturation protein 1 (Blimp1), which regulates eTregs activation and tissue homeostasis, is required for IL-10 production and ICOS expression (16). Interferon regulatory factor 4 (IRF4), one molecule expressed in both Th2 cells and Tregs, was demonstrated to be essential for Tregs to suppress Th2 responses and was further shown to be required in type 1 inflammatory conditions due to its role in the effector Treg differentiation (16, 17). *Irf4*^–/–^ Tregs show impaired CD62L downregulation and loss ICOS expression in the mixed chimeric mice generated with WT and *Irf4*^–/–^ bone morrow (16). Furthermore, JunB, an important AP-1 factor, has also been observed to promote ICOS expression in basic leucine zipper transcription factor ATF-like (BATF)-dependent and BATF-independent manners in murine eTregs (18). By chromatin immunoprecipitation (ChIP)-sequencing analysis, Koizumi et al. have demonstrated that JunB can facilitate DNA-binding of IRF4 at sites located near *Icos* (18). In addition, NF-κB also plays an essential role in Treg identity and function, among which two canonical subunits are c-Rel and RelA (p65). NF-κB c-Rel is critical for thymic Treg development, and it was also shown to be important for the function of activated Tregs, as a number of genes associated with homeostasis and function of aTregs were dramatically downregulated in c-Rel-deficient Tregs, whereas RelA (p65) mediates the development, survival, and function of eTregs (19–21). RelA-deficient Tregs were observed to show a significant reduction in the expression of eTreg signature genes, such as *Icos*, *Tigit*, and *Il10*, which share some similarities with IRF-4-deficient Tregs (21). However, RelA acts independently of IRF4 in the regulation of eTreg development and function. TNFRSF signaling, particularly GITR or TNF signaling, was demonstrated to activate RelA independent of TCR signals, thereby regulating Treg cell function (21).

In addition, it is intriguing that, regardless of IL-10, a small but significant subgroup of ICOS^+^ Tregs were shown to produce IFN-γ and IL-17 as well as the corresponding specific transcription factors T-bet and RORγt in normal human peripheral blood and in draining lymph nodes of DNFB-sensitized mice (10, 13). Thus, although most studies have defined ICOS^+^ Tregs as a subset of activated Tregs with high suppressive function, we still have some reservations regarding these IL-17 or IFN-γ producing Treg cells. One reason is that the identification of Tregs based on CD25 and FOXP3 expression cannot guarantee that all the identified cells are real Tregs. It is inevitable that a small group of activated effector T cells are included in CD25^+^FOXP3^+^ T cells, (22, 23) which could be IL-17 or IFN-γ positive, and this proportion could be even higher in inflammatory environment. Another reason is that the stability and function of IL-17 or IFN-γ producing Tregs remain unelucidated.

Recently, a series of studies have been done to investigate the specific identities of T-bet^+^/RORγt^+^ Tregs or IFN-γ^+^/IL-17^+^ Tregs in homeostasis and various disease conditions. T-bet^+^ Tregs have been demonstrated to be detectable in the steady state and show an increased frequency and absolute number during type 1 immune responses, thereby playing an important role in the maintenance of Treg homeostasis in the inflammatory environment (24, 25). Although T-bet^+^ Tregs could not represent a stable Treg subset in the steady state, as the expression of T-bet in Tregs can be easily influenced by cytokine environment *in vitro* and *in vivo*, thus displaying a highly dynamic pattern, (25) T-bet^+^ Tregs were shown to be necessary and capable of restraining the proinflammatory role of pathological T cells (26). Remarkably, only a small proportion of FOXP3^+^T-bet^+^ cells are IFN-γ-positive in the steady state (24). Similarly, RORγt^+^ Tregs can also be detected in lymphoid and non-lymphoid tissues of mice in the steady state (27). In particular, significantly higher RORγt expression was observed in colonic Tregs, which is probably induced by symbiotic microbiota (27–29). These colonic RORγt^+^ Tregs display a CD44^hi^CD62L^lo^ eTreg phenotype with absent expression of Helios or Nrp-1 and a low degree of demethylation in *Ikzf2* (Helios) site, indicating that they are derived from pTregs (27–29). Additionally, these colonic RORγt^+^ Tregs were shown to be relative stable, although they were observed to loss FOXP3 expression slightly higher than FOXP3^+^ Tregs when transferred to lymphopenic mice together with naïve T cells, and to be necessary to maintain colonic homeostasis, displaying a superior regulatory capacity to prevent colitis (27). Moreover, these colonic RORγt^+^ Tregs express IL-10 but few IL-17^+^ cells even in inflamed colon (27, 28).

However, consistent with the idea that strong self-antigen stimulation could promote the loss of FOXP3 expression in Tregs, thus favoring ex-Treg formation in inflammatory setting, (30) a large number of transferred CBir1-specific FOXP3^+^ Tregs in TCRβxδ^–/–^ mice that were induced *in vitro* by culturing naïve CD4^+^ T cells in the presence of TGF-β lost FOXP3 expression and converted to IFN-γ^+^/IL-17^+^ T cells, and a fraction of transferred cells obtained IL-17^+^/IFN-γ^+^ Treg phenotype (31). This phenomenon could also be partly explained by their use of *in vitro* induced Tregs (iTregs) in the experiments, as iTregs are less stable than tTregs to maintain FOXP3 expression due to conserved non-coding DNA sequence (CNS) 2 CpG island hypermethylation (32). Additionally, although these CBir1-specific Foxp3^+^IFN-γ^+^ T cells retained suppressive ability, it was observed that these FOXP3^+^IFN-γ^+^ T cells can differentiate into IFN-γ^+^ T cells ultimately (31). Similarly, CD4^+^FOXP3^+^IL-17A^+^ T cells in the dermis of lesional skin and an enhanced propensity of purified peripheral blood Tregs to convert to IL-17A^+^ T cells after *ex vivo* stimulation compared with healthy individuals have been shown in severe psoriatic patients (33). Therefore, it is hard to say whether the stability and the inhibitory capacity of ICOS^+^IFN-γ^+^/IL-17^+^ Tregs that are observed in both the steady state and inflammatory condition are still retained. Activated eTregs have been shown to be less stable than cTregs to maintain FOXP3 expression and ICOS-induced higher PI3K signaling also contributes to this instability (34, 35). The stability and function of these specific ICOS^+^ Tregs need further detailed inspection.

### The Role of ICOS Signaling in Tregs

According to various studies, ICOS is generally involved in the production, proliferation and survival of Tregs, providing them a strong suppressive capability, which we will discuss separately below.

First, ICOS can mediate the generation of FOXP3^+^ Tregs. Compared with healthy individuals, CD4^+^CD25^–^ T cells from a small subset of common variable immunodeficiency (CVID) patients who have a homozygous genomic deletion of ICOS cannot induce anergic T cells with immature myeloid DCs, (36) which are involved in maintaining peripheral tolerance by the induction of Tregs (37). Furthermore, the ablation of ICOS in unmanipulated mice was shown to result in a reduced number of FOXP3^+^ Tregs compared to that observed in WT mice (38, 39). Blockage of ICOS-ICOSL ligation when culturing human naïve CD4^+^ T cells with CD40-activated B cells *in vitro* was also shown to decrease FOXP3 levels, thereby impairing the generation of FOXP3^+^ Tregs (40). These phenomena confirm the importance of ICOS for the transcriptional activity of *Foxp3*, which could be illustrated by favoring the combination of nuclear factor of activated T cells (NFAT) to FOXP3 over NFAT to activator protein 1 (AP-1) (41). In addition, ICOS-deficient Treg cells showing Foxp3 instability due to significant methylation of *Foxp3* CNS2 may be another reason for the reduction of Tregs (14). However, the reduced frequency of FOXP3^+^ Tregs in the periphery should not be attributed to the dysfunction of the thymus, as *Icos*^–/–^ mice exhibit a similar percentage of FOXP3^+^ T cells in the thymus compared with WT mice (38). In contrast, by analyzing TCR excision circles, a significant increase in the thymic output of Tregs was detected in *Icos*^–/–^ mice compared with that observed in WT mice (14).

Second, the ICOS signal aids in promoting the proliferation of Tregs. The superior proliferative capacity of ICOS^+^ Tregs has been confirmed by a higher Ki-67 expression expressed on these cells (42, 43). ICOSL expressed on plasmacytoid DCs was demonstrated to preferentially promote ICOS^+^ Treg proliferation by engaging with ICOS on Tregs *in vitro* (9). When immunizing *Icosl*^–/–^ mice with endotoxin-free ovalbumin (OVA), which is known to facilitate Treg proliferation, the proportion of FOXP3^+^ antigen-specific Tregs was significantly lower than that observed in WT mice, suggesting a great effect for ICOS co-stimulation in the expansion of Tregs (38). Likewise, a delayed and insufficient Treg expansion was also observed in C57BL/6 *Icos*^–/–^ mice during helminth infection (44). Nevertheless, the results of assays using BrdU to track T cell proliferation indicated that no difference in BrdU uptake was observed in FOXP3^+^ Tregs from *Icos*^–/–^ mice upon infection compared with that observed in WT mice. The deficiency of Tregs could result from the enhanced apoptosis of *Icos*^–/–^ Tregs (44). From this perspective, the auxo-action of ICOS to Treg proliferation and survival is hard to clearly measure.

Indeed, ICOS signaling is highly involved in the survival of Tregs. When murine CFSE-labeled Tregs were stimulated with anti-CD3 antibody for 2 days, ICOS^–^ Tregs died within the few hours after TCR stimulation, while ICOS^+^ Tregs became hyperproliferative, suggesting a death tendency of Tregs with absent ICOS expression (43). Furthermore, during the *in vitro* stimulation culture in the presence of IL-2, purified mouse ICOS^+^ Tregs from lymph node and spleen expressed a higher level of Bcl-2, an anti-apoptotic molecule belonging to the Bcl-2 super family, than ICOS^–^ Tregs (42). But the higher expression of Bcl-2 in ICOS^+^ Tregs could also be partly explained by the addition of IL-2, as Bcl-2 is known to be sensitive to IL-2 stimulation, and ICOS^–^ Tregs do not respond to IL-2 like ICOS^+^ Tregs and therefore do not upregulate Bcl-2 expression (42). Indeed, CD44^hi^CD62L^lo^ eTregs that express high levels of ICOS exhibit low Bcl-2 expression under steady state conditions, and these Bcl-2^lo^ eTregs were observed to be selectively lost after 2 weeks of ICOSL blockage in WT mice, indicating an important anti-apoptotic role of ICOS signaling in promoting Bcl-2^lo^ eTregs survival (12).

However, Bcl-2 is not exclusively important for Treg survival. There are many other molecules belonging to the Bcl-2 family involved in the regulation of Treg survival, especially Bim and Mcl-1. Chougnet et al. reported that Tregs accumulation in aged mice could be attributed to the increased survival due to decreased expression of Bim in Tregs, while neither Bcl-2 nor Mcl-1 contributes to the increased survival in old Tregs (45). It was demonstrated further that the reduced Bim expression in Tregs mainly promotes effector, but not central, Tregs accrual, as the accumulated Tregs in aged mice are mainly CD44^hi^CD62L^lo^ eTregs, and Treg-specific or germline deletion of Bim cannot rescue the decreased number of cTregs with age in mice (46). Moreover, Mcl-1, but not other anti-apoptotic proteins including Bcl-xl and Bcl-2, has been demonstrated to be particularly important for the survival of Tregs to maintain homeostasis (47).

These two essential proteins have also been shown to be included in ICOS-mediated survival signals. By comparing the expression levels of ICOS on Tregs in mice of different ages, Raynor et al. showed a coordinating increase in ICOS expression with the age-dependent accrual of regulatory T cells in old mice and indicated an effect of ICOS/ICOSL interaction on Tregs homeostasis, which is mediated by antagonizing Bim in eTregs (46). The increased levels of IL-6 that occurs with age enhanced TCR-driven ICOS expression, thereby attenuating Bim expression and sustaining aged effector Tregs survival, possibly though PI3K/Akt/FOXO pathway (46). Moreover, ICOS-ICOSL interaction could yield the recruitment of PI3K components, leading to cross activation of the PI3K-Akt downstream signals, which was previously suggested to mediate anti-apoptotic effects. For instance, blocking p110δ with the selective inhibitor CAL-101 can lead to sufficient GSK-3β activation, resulting in the degradation of the anti-apoptotic protein Mcl-1 and effectively impeding Tregs survival (48). In summary, ICOS signaling can mediate Treg survival by fine-tuning the expression of multiple anti-apoptotic and pro-apoptotic molecules of Bcl-2 family.

In addition, one cytokine that is worth mentioning is IL-2, which is also regarded as being essential for the expansion and survival of Tregs. IL-2 signaling was shown to induce FOXP3 expression during the Treg development in the thymus and was observed to be widely involved in Treg differentiation, lineage stability, proliferation, and function (49). Phosphorylation of STAT5 could be an important hallmark for the activation of the IL-2 signals. It was demonstrated that pSTAT5^+^ Tregs exist as discrete clusters in secondary lymphoid tissues of mice with IL-2-producing proto-effector T cells and DCs to exert suppressive functions, making effects to maintain immune homeostasis (50). Moreover, some survival factors that can be regulated by ICOS have also been observed to be regulated by IL-2. For example, IL-2 addition can increase Mcl-1 expression *in vitro* and *in vivo* (47). Here, we would like to discuss the effect of IL-2 to ICOS^+^ Tregs and the non-redundant role of IL-2 and ICOS signaling in Treg biology.

As is reported by Kornete et al., the effect of IL-2 to ICOS^+^ Tregs is much more than the superficial cognition that it promotes Tregs expansion, IL-2 gives a functional fitness to ICOS^+^ Tregs (42). In other words, ICOS^+^ Tregs are more sensitive to IL-2 stimulation and more dependent on IL-2 to maintain their survival than ICOS^–^ Tregs. They observed a higher responsiveness to IL-2 in ICOS^+^ Tregs, as a fold increase of surface marker CD25, a high-affinity IL-2 receptor component, and STAT5 phosphorylation were detected in ICOS^+^ Tregs when culturing Tregs from BCD2.5 Foxp3^GFP^ mice with APCs (42). Furthermore, the withdrawal of IL-2 led to an apoptotic tendency of ICOS^+^ Tregs during the culturing of separated ICOS^+^ Tregs and ICOS^–^ Tregs, which could be rescued by re-adding IL-2 (42). This result was not only observed in mice, but has also been reproduced in human cells, indicating an important role of IL-2 in the survival of ICOS^+^ Tregs *in vitro* (9).

However, it should be noted that ICOS may promote Treg proliferation or survival independently of IL-2 signaling *in vivo*. On one hand, the ablation of ICOS was observed to reduce the inhibitory function of Tregs without affecting their reactivity to IL-2, as *Icos*^–/–^ BDC2.5 Tregs exhibited no difference with WT Tregs in their intrinsic responses to IL-2, (42) and transferring OT-II T cells into *Icosl*^–/–^ mice did not affect IL-2 production by these T cells (38). On the other hand, the results of a study using C57BL/6 mouse model have indicated that blockage of IL-2 signaling results in significant loss of CD44^lo^CD62L^hi^CCR7^hi^ cTregs without significantly impacting the number or the proportion of CD44^hi^CD62L^lo^CCR7^lo^ eTregs, which express high levels of ICOS, in the lymphoid or non-lymphoid tissues (12). Moreover, although a great increase of CD25 expression was observed in ICOS^+^ Treg after IL-2 stimulation *in vitro*, eTregs express a lower level of CD25 and less depend on IL-2 than cTregs for their maintenance *in vivo* (12). The signaling switch from IL-2 to ICOS in eTregs is probably due to the unique environment where eTregs are located, as they mostly populate in peripheral tissues, in which IL-2 is not prevalent, whereas cTregs are continuously circulating between lymphoid tissues and blood and could easily gain access to IL-2 in the T cell zones of secondary lymphoid organs (12). In addition, Zhang et al. further demonstrated that this signaling switch in eTregs can cause FOXP3 instability, consistent with the opinion that a small fraction of activated Tregs could loss FOXP3 expression and transform into Th cells under disease conditions (34).

Last but not least, ICOS endows Tregs with a stronger suppressive function. Recent many studies have shown the significance of the ICOS-ICOSL signaling pathway for the self-tolerance mediated by Tregs. *Icos*^–/–^ mice, which fail to develop respiratory tolerance induced by intranasal Ag application, display no significantly increase in splenic and lung-resident CD4^+^FOXP3^+^ Tregs after being immunized with OVA and a decreased ability to secrete IL-10 compared with that observed in WT mice (39). Similarly, ICOS^+^ Tregs were suggested as a dominant Treg subset to prevent NOD mice from the development of diabetes, and a drastic reduction in ICOS expression on pancreatic Tregs was observed as NOD mice progressed from prediabetic stage to overt diabetes (42).

The stronger inhibitory ability of ICOS^+^ Tregs than ICOS^–^ Tregs was partly relies on the higher expression levels of IL-10. Indeed, as early as in 2003, Löhning et al. observed that ICOS^high^ T cells were closely correlated with the expression of IL-10 (8). Soon afterward, ICOS-dependent IL-10 production was demonstrated to be indispensable for the acquisition of the suppressive ability of T cells generated in DO × OVA^high^ mice, which presented a self-tolerance phenotype with low numbers of T cells and a reduced proliferative ability compared to DO × OVA^low^ mice, owning to the largely generated IL-10-producing CD4^+^ T cells (51). Furthermore, ICOS^+^ Tregs and ICOS^–^ Tregs were shown to exert inhibitory function through different molecular mechanisms. ICOS^+^ Tregs produce higher amount of IL-10 but lower level of TGF-β than ICOS^–^ Tregs. By adding a neutralizing antibody against IL-10 or blocking TGF-β signaling with a pharmacological inhibitor when culturing ICOS^+^ or ICOS^–^ Tregs with naïve T cells *in vitro*, the inhibitory function ICOS^+^ Tregs was shown to be mediated through both IL-10 and membrane TGF-β (mTGF-β), whereas ICOS^–^ Tregs only used mTGF-β in the cell-cell contact dependent manner (9).

However, although some studies suggested a correlation between ICOS expression and IL-10 secretion, other researchers observed no reduction of IL-10 production when ICOS ligation was blocked. For example, ICOS deficiency resulted in a large reduction in FOXP3^+^ Tregs in the spleen and colonic lamina propria but a similar level of IL-10 expression relative to that observed in *Icos*^+/+^ mice (14). In addition, IL-10 was shown to be unnecessary for ICOS-mediated suppressive function in some cases. The intranasal application of Protollin inhibited allergen-induced airway hyperresponsiveness (AHR) through toll-like receptor (TLR) 4 dependent ICOS^+^ Tregs induction (52). However, IL-10 was demonstrated to be dispensable for its inhibition of experimental asthma, although increased *Il10* mRNA could be detected in nasal associated lymphoid tissue (NALT) harvested from mice 14 days after Protollin administration (52). These conditions not only indicate the possibility that there are other IL-10-producing CD4^+^FOXP3^–^ T cells that can compensate the reduced secretion of IL-10 induced by ICOS signaling in its absence, more importantly, there are also other ways for ICOS to mediate the inhibitory function of Tregs. In other words, the part of ICOS signaling that mediates the suppressive function of Tregs incompletely overlaps with the IL-10 signals. As an example, blockage of IL-10R or ICOSL was observed to cause divergent outcomes to mice with chronic *toxoplasma gondii* infection (53). Treatment with anti-IL-10R blocking Ab increased CD4^+^FOXP3^–^ effector T cell expansion, activated APCs, recruited numerous neutrophils to the brain and led to an eventually fatal immunopathology, whereas blocking ICOS signaling only resulted in a non-lethal expansion of T cells without limited IL-10 expression or APC activation (53). Additionally, IL-10-deficient mice develop more severe disease than *Icos*^–/–^ mice (38, 54). Although B and T lymphocytes are generated normally in both knockout mice, germline deficiency for *Il10* leads to development of chronic enterocolitis spontaneously, whereas *Icos* ablation does not (38, 54). Using Treg-specific IL-10 knockout mice, it was further shown that Treg-derived IL-10 production can help to keep immune responses in check at mucosal interfaces (55).

**FIGURE 1:**
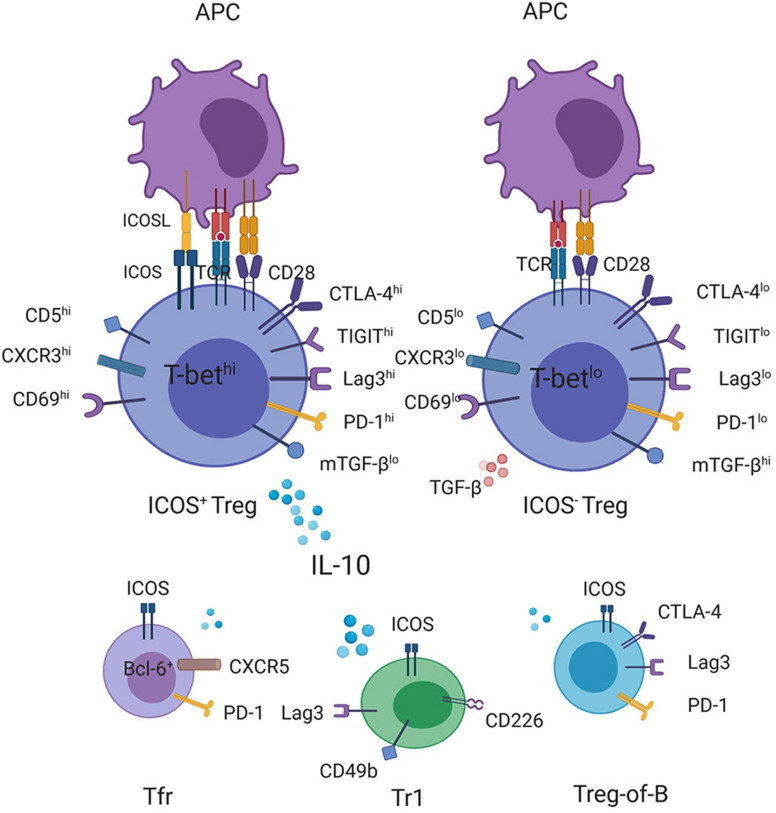
Production of IL-10 by ICOS-expressing T cells with regulatory function. CD4^+^ICOS^+^ Tregs express high levels of IL-10 but smaller amounts of TGF-β compared with ICOS^–^ Tregs. Transcriptome and FACS analyses have demonstrated a correlation between the increased ICOS expression on Tregs with the high expression levels of CD5, CD69, CXCR3, and T-bet as well as some checkpoint inhibitors, such as CTLA-4, TIGIT, Lag3, and PD-1, (15, 56, 57) some of which have been shown to promote the transcription of the IL-10 gene, contributing to high levels of IL-10 secretion together with ICOS. In addition, other ICOS-expressing regulatory cells, such as Tr1, Treg-of-B cells, and Tfr cells, can also be the source of IL-10.

At present, the mechanism underlying the ICOS-associated suppressive function of Tregs is still unclear. For one thing, it remains confusing how the downstream pathways of ICOS ligation induce *Il10* transcription. Recently, the results of many studies have confirmed the correlation between the increased expression of ICOS and the overexpression of CTLA-4, GITR, lag3, TIGIT, and CD69 on Tregs, (9, 15, 56, 57) and some of these upregulated markers have been shown to be involved in exerting the suppressive function of Tregs through IL-10 induction (Figure 1) (58, 59). For example, CD69^+^ Tregs, which highly express ICOS, were shown to be unable to exert their inhibitory function in *Il10* knockout mice (57). Similarly, compared with TIGIT^–^ Tregs, TIGIT^+^ Tregs expressed higher amounts of ICOS and other co-inhibitory molecules, including CTLA-4, PD-1, Lag3, and Tim3, and exhibited high levels of IL-10 expression, endowing Tregs inhibitory capabilities (15). However, despite TIGIT ligation induced *Il10* gene expression *in vitro*, IL-10 was not detectable in culture supernatants of TIGIT agonist-treated Tregs, which was isolated from immunized mice and was restimulated for 2 days *in vitro* (15). Additionally, deletion of Fgl2, an important mediator for TIGIT-mediated Treg suppressive function, did not affect TIGHT-mediated IL-10 induction in *Fgl2*^–/–^ Tregs *in vitro*, indicating that the TIGIT-related IL-10 pathway diverges from TIGIT-CEBPα-Fgl2 inhibitory signaling pathway (15). Therefore, the upstream signaling pathways of IL-10 as well as the interaction between ICOS and these molecules that promote IL-10 secretion require further study. For another thing, the existence and transmission mode of the ICOS-related but IL-10 independent suppressive signals remain an enigma. In addition to the direct inhibition mechanism mediated by ICOS, an enhanced migration of Tregs to target organs mediated by ICOS could also contribute to their increased inhibitory function. Co-stimulation with ICOS was shown to downregulate CD62L and CCR7 while upregulating many chemokine ligands on CD4^+^ T cells, which led to reduced homing capacity to lymph nodes but increased accumulation of Treg cells in target tissues (60, 61). For example, using photoconvertible fluorescent proteins to track Tregs recirculating between inflamed colon and the distal part of mesenteric lymph nodes, these migratory Tregs were discovered to display a highly immunosuppressive phenotype with a high expression of ICOS, Lag3, CTLA-4, CD103, PD-1, and CCR5 while exhibiting reduced expression of CCR7 (62). Intestinal inflammation increased Treg turnover as well as the number of Tregs with an inhibitory phenotype mentioned above, which contributed to controlling dextran sodium sulfate (DSS)-induced colitis (62). Additionally, except for ICOS^+^ Tregs, Tr1 cells, Tfr cells, and B-cell induced CD4^+^ Foxp3^–^ regulatory T cells (Treg-of-B cells), which also highly express ICOS, can exert suppressive abilities *in vitro* and *in vivo* (6, 63).

### PI3K Signaling and ICOS in Tregs

**FIGURE 2:**
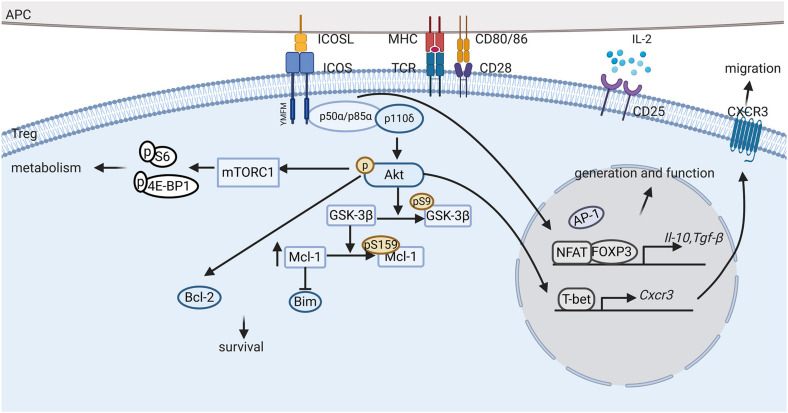
ICOS signaling pathway in Tregs. ICOS-ICOSL interaction promotes the generation, proliferation, survival and suppressive ability of regulatory T cells (Tregs) through complex signaling pathways. First, activation of ICOS promotes *Foxp3* transcription, favoring NFAT binding to FOXP3 over AP-1 and upregulating FOXP3 downstream regulatory genes, such as *Il-10* and *Tgf-*β. Second, ICOS engagement induces PI3K recruitment to the YMFM motif at the cytoplasmic tail and the phosphorylation of Akt. Activation of Akt can induce Bcl-2 expression and inhibit pro-apoptotic Bcl-2 family protein production, thereby promoting ICOS^+^ Treg survival. Furthermore, ICOS also activates the mTORC1 signals, which is suggested to mediate protein synthesis and metabolism in Tregs. In addition, ICOS expression elevates CXCR3 expression, which promotes the migration of Tregs to inflammatory tissues.

The most thoroughly studied downstream signaling pathway in ICOS^+^ Tregs is the PI3K signal pathway. The class I_A_ phosphatidylinositol 3-kinase (PI3K), which is widely involved in relaying signals from TCR and co-stimulatory receptors of T cells, are heterodimeric enzymes made up of a regulatory subunit (p85α, p55α, p50α, p85β or p55γ) and a catalytic subunit (p110α, p110β, or p110δ) (64). Although there is no difference in the expression levels of p110α, p110β, or p110δ in Tregs and Tcons, p110δ plays an indispensable role in Treg cells (48). ICOS cross-linking results in the phosphorylation of Tyr^181^ in the YMFM motif, which binds to the SH2 domain of p85α or p50α, and further recruits the p110 catalytic subunit to activate downstream molecules, such as the lipids phosphatidylinositol (3, 4)-biphosphate (PIP2) that is subsequently converted to phosphatidylinositol 3,4,5-trisphosphate (PIP3) at the inner leaflet of the cytomembrane under the catalysis of p110 (64). After that, PIP3 phosphorylates Akt, which activates complex downstream signals, thus playing an essential role in Treg cell proliferation, survival as well as metabolism, some details of which are discussed in the preceding section (Figure 2).

In addition, the PI3K-mTORC signal is also involved in the regulation of ICOS in Tregs. As an intracellular energy metabolism receptor, the mammalian target of rapamycin (mTOR), which is comprised of mTOR complex 1 (mTORC1) and mTORC2, integrates immune and metabolic signals inside and outside T cells, participates in the maintenance of T cell homeostasis, and determines the fate of cells (65). Under steady state conditions, Tregs display elevated phosphorylation of two major substrate molecules downstream of mTORC1, 4E-BP1 and S6, compared with naïve T cells (66). Although numerous studies have revealed a negative effect of the mTORC signaling on the *de novo* differentiation and population expansion of Tregs, (67, 68) and mTORC1 and mTORC2 are likely to mediate the inhibition of Treg differentiation through different mechanisms, (65) mTORC1 is suggested to be responsible for the homeostasis and suppressive activity of Tregs (66). Zeng et al. demonstrated that selective deletion of Raptor in Tregs caused profound inflammatory diseases accompanied by lymphoproliferative manifestation in *Foxp3*^cre^*Raptor*^fl/fl^ mice, despite these Raptor-deficient Tregs exhibited normal FOXP3 expression and retained the capacity to produce the anti-inflammatory cytokines, TGF-β1 and IL-10 (66). Whereas, Raptor-deficient Tregs showed decreased expression of CTLA-4 and ICOS, two important molecules for Treg function, and impaired proliferation ability in a disease-free environment. Furthermore, they suggested that Raptor/mTORC1 signaling can regulate cholesterol/lipid metabolism through the mevalonate pathway, promoting enhanced Treg proliferation and CTLA-4 and ICOS upregulation, which increase Treg function (66). In addition, despite the mTORC1 signaling have been demonstrated to be essential to the homeostatic proliferation of Tregs, Xu et al. reported that a half reduction of mTORC1 signaling in Tregs did not apparently influence the expansion and suppressive function of FOXP3^+^ Tregs in *Raptor*^fl/+^*Foxp3*^Cre^ mice after OVA/CFA immunization (69). In contrast, a decrease in the percentage and absolute number of FOXP3^+^CXCR5^+^ Tfr cells was observed in *Raptor*^fl/+^*Foxp3*^Cre^ mice compared with that observed in WT mice, and these *Raptor*^fl/+^ Tfr cells also expressed a decreased level of CTLA-4, ICOS, and PD-1 (69). Indeed, the mTORC1 signaling is critical for both the *de novo* differentiation of Tfr cells from conventional Tregs precursors by activating mTORC1-p-STAT3-TCF-1-Bcl-6 axis and their suppressive functions (69). Moreover, except for the fact that mTORC1 signaling could promote ICOS expression in Tfr cells, ICOS signaling also promotes Tfr cell differentiation and function in immunized mice (70).

The PI3K-mTORC2 signaling also plays an essential role for Tregs differently from mTORC1 signaling. PETN-mTORC2 can regulate metabolic balance between glycolysis and mitochondrial fitness in Tregs which is probably associated with Treg stability (71). Compared with effector T cells, Tregs maintain high levels of PTEN to control PI3K signal strength at an appropriate level, which is essential for Tregs to maintain lineage stability and homeostasis (35). This condition can also be supported by the fact that activated eTregs, which upregulate ICOS expression and activate PI3K signaling, can be less stable than cTregs (34). Deletion of PTEN disables Treg to maintain an activated phenotype and fails to create a suppressive tumor microenvironment, therefore displaying a protective role in tumor models (72). *Pten*-ΔTreg mice, which specifically delete PTEN in FOXP3^+^ Tregs, develop an autoimmune-lymphoproliferative disease featured by uncontrolled Th1 and Tfh responses and excess germinal center formation (35, 71). PTEN deletion causes great Treg proliferation and higher expressions of CD44, CD69, ICOS, and PD-1 but a lower expression of CD62L in *Pten*^fl/fl^*Foxp3*^Cre^ Tregs, indicating an activated phenotype (35, 71). However, these PTEN-deficient Tregs are unstable, which could spontaneously downregulate CD25 and subsequently loss FOXP3 expression during *in vitro* culture with IL-2 supplementation and as *Pten*-ΔTreg mice age, and might be pathogenic as they could not resolve the induced experimental autoimmune encephalomyelitis in *Pten*-ΔTreg mice (35). The loss of function of PTEN-deficient Tregs could be restored by inhibition of mTORC2 signaling that is upregulated after PTEN deficiency (71). In addition, PTEN-mTORC2 axis has also been shown to be important for Tfr cells to suppress Tfh and germinal center responses (71).

## Similarities and Differences Between ICOS and Other CD28 Family Members

### ICOS and CD28

As a member of the CD28 family, human ICOS is a type-I transmembrane protein that shares 24% identity with CD28 and 17% identity with CTLA-4 (1). Previous studies have reported a synergistic co-stimulatory effect of ICOS and CD28 to promote T cell activation, proliferation and function. However, there are still some differences in the downstream signals between ICOS and CD28, and they function in somewhat different ways.

ICOS possesses the YMFM motif at the cytoplasmic tail, which is the YMNM motif at the corresponding site of CD28 (73). The YMFM motif allows ICOS to preferentially recruit p50α that has a stronger lipid kinase activity than p85α, therefore activating PI3K more strongly than CD28 (74). Transformation of asparagine (N) to phenylalanine (F) in ICOS also causes it to lose the ability to bind with the SH2 domain of Grb2 that activates the NFAT/AP-1 site to promote IL-2 transcription (75). Furthermore, different abilities to activate MAPK have been observed, with CD28 exhibiting a stronger ability to phosphorylate p46 JNK (76). Moreover, the absence of an MYPPPY motif in the single immunoglobulin (lg)V-like extracellular domain inhibits the ability of ICOS to bind to B7-1/B7-2 like CD28 (77). Alternatively, ICOS interacts with a unique ligand ICOSL, which is also expressed on ILCs and some types of non-hematopoietic cells except for APCs, increasing its functionality. Additionally, upon TCR stimulation, ICOSL can be rapidly shed after binding to ICOS to maintain a proper strength of the co-stimulatory signal on T cells, but the same situation has not been observed in CD28-B7-1/B7-2 interaction (78, 79). This process is demonstrated to be mediated by the AMDM family-dependent proteolytic cleavage of ICOSL on B cells (80).

These differences between ICOS and CD28 signaling could lead to different effects on Tregs. It has been well established that CD28 plays an essential role in the development, proliferation, and function of Tregs. *Cd28*^–/–^ mice display a significant reduction of Tregs in the spleen and other secondary lymphoid organs compared with WT mice (81). Remarkably, CD28 is important for the development and homeostatic proliferation of tTregs, whereas the anti-apoptotic activity or slow proliferation in the steady state of pTregs was observed unaffected by CD28 deficiency (81). Wakamatsu et al. have also shown that pTregs were still abundant in the intestines of *Cd28*^–/–^ mice, although a weaker suppressive ability of these *Cd28*^–/–^ Tregs was observed, suggesting that pTregs can be developed in a CD28-independent manner (82). Additionally, during the *in vitro* generation of iTregs, CD28 stimulation was considered to be a limiting factor for the acquisition of Treg-specific DNA hypomethylation at the signature genes of Tregs (83). In contrast to CD28, ICOS deficiency seems not to impair thymus output of Tregs, although the number of Tregs was observed to be reduced by 30% in *Icos*^–/–^ mice (14, 38). Therefore, ICOS signaling may be more involved in Treg generation in the periphery and eTreg expansion and function. But it could not be excluded that the expression of tTreg signature genes is affected during the development in the thymus when ICOS signaling is absent, which should be clarified further. Furthermore, when isolated ICOS^+^ Tregs were cultured in the presence of anti-CD3 mAb and IL-2, the addition of ICOSL was observed to facilitate Treg proliferation, whereas adding anti-CD28 mAb plus ICOSL strongly inhibited this promotion (9). This result not only indicates a negative effect of CD28 to promote ICOS^+^ Treg proliferation *in vitro*, but also emphasizes a proliferation promoting effect of ICOS independent of CD28. In addition, ICOS signaling induces the production of large amounts of IL-10 but limited IL-2 secretion, just opposite to CD28 signaling (76).

### ICOS and CTLA-4

Unlike CD28 which is constitutively expressed on the surface of T cells, or ICOS which exhibits an inductive expression pattern, CTLA-4 is rarely detected on resting T cells but is predominately centralized in the intracellular compartments of Tregs and activated T cells (84). Only under specific conditions can CTLA-4 be expressed on the cell surface. CTLA-4 interacts with B7-1/B7-2 with higher affinity and avidity than CD28 and plays the opposite role to CD28. Unlike *Icos*^–/–^ mice that remain healthy and have normal absolute number of lymphocytes in homeostasis, *Ctla-4*^–/–^ mice develop fatal lymphoproliferative disease, characterized by splenomegaly, lymphadenopathy, multiorgan lymphocytic infiltration, and tissue destruction (38, 85, 86). Hyperproduction of lgE and lgG reveals the susceptibility to autoimmune diseases of CTLA-4 deficient mice (86, 87). Similar but less severe symptoms are also observed in mice with Treg-specific CTLA-4 deficiency, indicating that this disorder is largely attributed to loss of function of CTLA-4 deficient Tregs to suppress T cell responses (87). Additionally, Wing et al. have also demonstrated that CTLA-4 mainly affects Treg suppressive function in the periphery, the generation of FOXP3^+^ thymocytes is minimally altered in CTLA-4 conditional knockout mice (87).

Interestingly, CTLA-4 has been observed to be expressed at relatively higher levels on the surface of ICOS^+^ Tregs, (9) suggesting a subtle connection between these two molecules that belong to the same superfamily. However, considering that CTLA-4 deficiency, but not ICOS, is associated with the loss of immune tolerance, there raises a question of whether the superior suppressive ability of ICOS^+^ Tregs relies on CTLA-4 expression. Recently, Zheng et al. reported that blockage of the ICOS-ICOSL interaction decreased the surface expression of CTLA-4 in CD4^hi^ Tregs induced by coculturing human naïve CD4^+^CD45RO^–^CD25^–^ T cells with CD40-activated B cells without impairment of total CTLA-4 production, and they ascribed the observed reduction to the strong recruitment of PI3K to the ICOS cytoplasmic domain, which competed with adaptor protein 2 (AP-2) to preferentially bind to mCTLA-4 (40). AP-2 is a critical factor to mediate CTLA-4 rapid endocytosis by targeting the YVNM motif on the cytoplasmic tail of CTLA-4, reduced combination of which with CTLA-4 retains more CTLA-4 proteins on the cell membrane (88). Additionally, ICOS has been suggested to be a pivotal marker of Tregs with a high capability to mediate CTLA-4-dependent transendocytosis, which is an important mechanism by which the activation signal can be attenuated by removing B7-1/B7-2 from opposing cells (89). In summary, ICOS expression plays a synergistic role for CTLA-4 to exert an inhibitory function of Tregs. The superior suppressive ability of ICOS^+^ Tregs is likely owned to the higher CTLA-4 expression in part. Additionally, we have to note that as a co-inhibitory molecule, CTLA-4 can effectively inhibit ICOS-producing effector T cells, complicating the links between ICOS and CTLA-4 in fine-tuning the immune responses in diseases as well as under steady state conditions.

Recently, immune checkpoint therapy has demonstrated great clinical efficacy in the areas of anti-tumor treatment. Blocking the negative regulator of co-stimulation CTLA-4 is a feasible means by which to disrupt the immunosuppressive microenvironment in tumor tissue. The anti-CTLA-4 monoclonal antibody ipilimumab has been confirmed to have efficacy in treating multiple types of advanced tumors, such as gastric cancer, non-small cell lung cancer (NSCLC), metastatic melanoma, and urologic neoplasms. Furthermore, an increased proportion of IFN-γ-producing CD4^+^ICOS^+^ T cells was sequentially observed in peripheral blood and tumor tissue after patients received anti-CTLA-4 therapy for bladder cancer, prostate tumors and metastatic melanoma, which was closely associated with clinical benefits and could serve as a specific pharmacodynamic indicator to monitor the efficacy of anti-CTLA-4 therapy (90–93). The benefits of this immunotherapy are a result of ICOS-activated PI3K signaling and T-bet expression that drives IFN-γ secretion and changes the tumor suppressive immune microenvironment to a Th1 cell-dependent anti-tumor immune state (94). Based on this evidence, an anti-CTLA-4 antibody and ICOS agonist combination treatment may be an even better means of treating cancer. In addition, the frequency of CD4^+^ICOS^+^ T cells was also shown to be unexpectedly elevated in non-tumor tissues during treatment with CTLA-4 antibody, causing immune-related adverse events, such as colitis, which should be improved in the future (91, 95).

## ICOS^+^ Tregs and Immune Diseases

The expression of ICOS has been observed to be upregulated in many autoimmune diseases, allergic diseases and different types of neoplasms. In some diseases, the upregulation of ICOS was most prominent in Tregs, and these ICOS^+^ Tregs displayed a pivotal function in these diseases. Here, we briefly introduce the role of ICOS^+^ Tregs in immune tolerance using several typical immune diseases as examples.

### ICOS^+^ Tregs and Type 1 Diabetes

Type 1 diabetes (T1D) is a common autoimmune disease induced by T cell-dependent damage to insulin-producing islet beta cells. The NOD mouse strain is a classical model of autoimmune diseases that can spontaneously develop autoimmune diabetes and highly simulates the pathogenesis of T1D in human subjects (96). Through over 10 years of studies of diabetogenesis in NOD mice as well as type 1 diabetic patients, researchers have shown that the breakdown of immunological tolerance is a crucial step in the development of this disease, with Tregs being a major component mediating immunosuppression. Functional waning of Tregs in aged NOD mice was shown to result in an insufficient ability to control pathogenic Teff infiltration within pancreatic sites, leading to diabetogenic insulitis lesions and beta cell damage, although the number of nTregs did not decrease relative to that observed in T1D-resistant mice (97). The ineffectiveness of Tregs has also been confirmed in humans by observing their deficiency in suppressing Teff proliferation *in vitro*, with similar proportions of CD4^+^CD25^+^FOXP3^+^ Tregs detected in age-matched healthy individuals and type 1 diabetic subjects (98, 99). Due to the observed reduction in quality rather than quantity of Tregs, researches therefore wondered if there are differences in Tregs composition between T1D subjects and controls. Finally, after a great deal of investigation, T1D progression was shown to be correlated with the decrease in ICOS expression by intra-islet Tregs (42). ICOS^+^ Tregs, in contrast to ICOS^–^ Tregs, were shown to be more proliferative and suppressive *in situ*, with a higher capacity to secrete IL-10 after islet-Ag stimulation. ICOS deficiency or Ag blockage impairs the competitive fitness of Tregs and fails to protect NOD mice from the onset of T1D (42, 100). Furthermore, Kornete et al. observed that ICOS^+^ Tregs preferentially expressed CXCR3 in the pancreatic lymph node of prediabetic NOD mice, and this expression gave them a better migratory ability to home to β-islets (61). This discovery highlights the crucial role of ICOS from another perspective, as *Cxcr3*^–/–^ NOD mice developed diabetes earlier than WT mice due to a decreased potential of these *Cxcr3*^–/–^ Treg cells to migrate from pancreatic lymph nodes to β-islets (101). Indeed, ICOS signaling is indispensable for the expression of CXCR3 on Tregs. Tregs from *Icos*^–/–^ NOD mice only express limited level of CXCR3 (61). These CXCR3-expressing ICOS^+^ Tregs showed a Th1-like phenotype, with an increased expression of T-bet and IFN-γ as well as its receptor IFN-γR, which enables ICOS^+^ Tregs to respond to IFN-γ produced by effector T cells and provides Tregs with an enhanced inhibitory ability (61).

In light of all of the evidence indicating the tremendous protective function of Tregs, particularly those that express ICOS, two therapeutic strategies have been suggested in the pathogenesis of T1D, and we will discuss below.

Low dose IL-2 supplementation or *Il2* protective allelic variation was shown to be sufficient to release the local deficiency of IL-2 in pancreatic islets, increase the number of Tregs and rebalance the Treg-Teff cell ratio in inflamed islets, thereby preventing the onset or reversing established diabetes eventually (42, 102–104). This benefit of IL-2 therapy was partially contributed to by the augmented expansion of ICOS^+^ Tregs in response to IL-2, which also imparted these regulatory cells with a higher anti-apoptotic and inhibitory function by promoting the expression of Bcl-2 and other Treg-associated proteins (CD25, CTLA-4, and GITR).

Another hotspot in diabetic treatment is the adoptive transfer of Tregs. The administration of autologous CD4^+^CD25^high^CD127^low^ Tregs, which were isolated from the peripheral blood of the same individual and expanded *ex vivo* under polyclonal stimulation, to children with recent-onset of type 1 diabetes, dramatically increased C-peptide levels and decreased the dependency on exogenous insulin, demonstrating a safe and efficient therapy for T1D (105, 106). In addition, with the further development of this approach, researchers observed that Ag-specific Tregs could more efficiently suppress autoimmune diabetes than the blind amplified polyclonal Tregs in a NOD mouse model (107, 108). Recently, with the help of single-cell T cell receptor analysis and MHC tetramer staining, the specific antigens recognized by islet Tregs were finally discovered, among which, antigens corresponding to the top two islet Treg clones were insulin B:9-23 and proinsulin (109). This result also provides guidance for the selection of more effective Tregs for transfer. However, due to the rarity of Tregs specific to a particular islet antigen in the human Treg pool, obtaining enough Ag-specific Tregs is hardly realized in clinical practice as in animal experiments, where it is achievable to gain sufficient seed cells for expansion using TCR-transgenic mice. Therefore, a better means appears to be the use of the entire repertoire of islet-specific Tregs for expansion in actual treatment. Interestingly, by examining the distinct and restricted islet-specific Treg repertoire, researchers observed that the dominant clones highly expressed the TCR-dependent markers CD103, TIGIT, and ICOS, and markers of recent antigen exposure such as CD5 and Nur77 (109). Similarly, another study investigating a subset of tissue-specific Tregs with a high degree of self-reactivity, CD5^hi^ Tregs, suggested that these highly self-reactive Tregs could provide significant protection against the development of diabetes (56). These CD5^hi^ Tregs from infiltrated islets of NOD mice demonstrated an increased transcription of genes associated with TCR signaling, as well as those associated with an inhibitory function (*Ctla-4*, *Icos*, *Lag3*, and *Tight*), which seems to be highly associated with the characteristics of islet-specific Tregs (56). Therefore, it is not unreasonable to speculate that both methods identify the same functional Treg subset, which could provide a convenient means of obtaining therapeutic Tregs using a combination of several molecular markers for the treatment of diabetes, and even other autoimmune diseases. Additionally, before the use of adoptive cell transfer therapy, the stability of these Ag-specific Tregs should be taken into consideration, as Tregs may loss some stability after TCR activation and have a risk of transforming into pathogenic ex-Tregs (110). Perhaps adopting some approaches to promote Treg stability, such as using monoclonal antibody drugs to intervene Nrp1/PTEN/Foxo axis, prior to and/or following Treg adoptive transfer may be a good solution (110).

In summary, ICOS^+^ Tregs are the primary cells in pancreatic islets involved in the prevention of diabetes, and any measures to expand these anti-inflammatory cells would efficiently treat diabetes.

### ICOS^+^ Tregs and IBD

Inflammatory bowel disease (IBD) is a chronic inflammatory disease characterized by the disruption of mucosal barrier function due to the dysbiosis of gut microbiota and unrestrained inflammatory responses mediated by effector T cells. Recent evidence has suggested that Tregs have a major role in the immune homeostasis of intestine and in the prevention of the development of IBD. For example, the transfer of CD4^+^CD25^+^ Tregs into mice with established inflammatory bowel disease, induced by injecting CD4^+^CD45RB^high^ T cells into severe combined immunodeficiency (SCID) mice, relieved wasting disease symptoms as well as infiltration of inflammatory cells (111).

To date, many therapeutic approaches have been shown to be effective against colitis in mouse models, most of which function by promoting the expansion and function of intestinal resident Tregs, especially ICOS-expressing Tregs. For instance, the injection of anti-CTLA-4 mAb was shown to ameliorate trinitrobenzene sulfonic acid (TNBS)-induced colitis by increasing indoleamine 2,3 dioxygenase (IDO) expression and inducing IL-10-producing ICOS^+^ Treg expansion in the mesenteric lymph nodes and inflamed colon (112). Besides that, the administration of galectin-3, which is limited in the inflamed intestinal epithelium of IBD patients but abundant in serum successfully suppressed the intense colonic inflammation induced by DSS or in a CD4^+^CD25^–^ T cell transfer model (113). Careful inspection showed a superior suppressive function of Tregs induced by galectin-3, which promoted the expression of ICOS, PD-1, and FOXP3 on CD4^+^ T cells *in vitro* in a dose-dependent manner (113). More recently, a study reported an interesting result where the adoptive transfer of CD69^+^ Tregs could attenuate severe colitis in two mouse models (57). Furthermore, they suggested that the reason why CD69^+^ Tregs were more effective than CD69^–^ Tregs for the treatment of IBD was the high IL-10 production induced by CD69 in a c-Maf- and STAT3- dependent manner (57). Intriguingly, these CD69^+^ Tregs were resistant to Th17 polarization and exhibited high expression of the immunosuppression-associated markers CTLA-4, ICOS, CD38, and ICAM-1 (57). This outcome was consistent with the view that IL-10 secretion is associated with ICOS expression on Tregs. In addition, with increasing attention being paid to the intestinal microbiota, there is a great deal of evidence suggesting that many species of commensal microorganisms have a strong capability to affect the proliferation and function of colonic Tregs (114). Among these indigenous microorganisms, 46 mouse and 17 human strains belonging to Clostridia clusters IV, XIVa and XVIII have been shown to promote the accumulation and differentiation of Tregs in the colon lamina propria by providing a TGF-β- and IDO-enriched intestinal environment (115, 116). Oral inoculation of Clostridia strains attenuated the colitis of adult mice induced chemically or by the transfer of CD4^+^CD45RB^hi^ T cells, providing another potential for treatment for IBD by rectifying bacterial dysbiosis (115, 116). Notably, most of the Clostridia-induced Tregs were helios^–^RORγt^+^CTLA-4^hi^ICOS^+^IL-10^+^ Tregs.

Considering all of the above evidence, we speculate that ICOS could serve as a pivotal marker representing an indispensable subset of Tregs, at least those located in intestinal tissue, which are more suppressive and indicative of the positive outcome of IBD. Landuyt et al. have confirmed that ICOS^+^ but not ICOS-deficient Tregs could ameliorate active colitis, which is a strong corroboration of our point of view (14). Indeed, not only are ICOS^+^ Tregs capable of maintaining T cell tolerance, various FOXP3^–^ regulatory T cells, such as Tr1 cells and Treg-of-B cells, which also express ICOS, can protect mice from experimental colitis (117). However, although ICOS appears to be beneficial by promoting the inhibitory ability of Tregs to assist in the prevention of IBD attacks, it was highly expressed on activated CD4^+^ T cells after the onset of IBD (118). In addition, Kanai et al. have proved the therapeutic potential of anti-ICOS mAb in colitis induced by transfer of CD4^+^CD45RB^hi^ T cells to SCID mice (119). This paradox also gives us a warning that a comprehensive and careful measurement of the impact of ICOS on the overall immune environment, not just confined to Tregs or effector T cells, should be made before the selection of an ICOS agonist or antagonist for the treatment of one type of disease.

### ICOS^+^ Tregs and Chronic Airway Inflammatory Diseases

Elucidating the complicated interactions between various immune cells or between immune and structural cells and attempting to control the excessive immune responses and oversecretion of inflammatory mediators are key and difficult issues in the study of chronic airway inflammatory diseases, among which, chronic obstructive pulmonary disease (COPD) and asthma are two major diseases. Up to now, there was little knowledge regarding the importance of ICOS-related signals in COPD. Recently, we observed an increased proportion of ICOS in CD4^+^CD25^+^FOXP3^+^ Tregs of COPD patients, yet the details of the function of this molecule remains to be studied (120). Herein, we emphatically introduce ICOS-associated signals in asthma.

Asthma is an airway hyperreactive disease mediated by type 2 immunity characterized by airway eosinophilic inflammation, mucus hypersecretion, high type 2 cytokines secretion and increased lgE antibody titers. T cell tolerance is the primary mechanism protecting human from the development of this disease. Numerous recent studies have revealed that Tregs and ICOS-ICOSL signals are highly involved in the anti-inflammatory process against asthma (39, 121). The percentage of ICOS^+^ Tregs was shown to be increased in the lungs of OVA-tolerized mice, and they exerted a strong capacity to inhibit Th2-mediated immune responses (91). ICOS deficiency was observed to restrain the suppressive ability of Tregs in controlling asthma, as the transfer of *Icos*^–/–^ Tregs into OVA-sensitized recipients could not suppress the severe allergic phenotype (91). Moreover, compared to WT mice, *Icos*^–/–^ mice were unable to generate an equal number of FOXP3^+^ Tregs in the lungs, indicating a potent proliferation-promoting effect mediated by ICOS signaling other than enhancing the inhibitory ability of Tregs (91). More importantly, ICOS signaling was reported to be widely involved in the induction of respiratory tolerance. Blockage of the ICOS signaling pathway in the induction stage of the immune tolerance of OVA-challenged mice impaired the development of respiratory tolerance (39, 121). This effect of the induction of tolerance was partly attributed to lung plasmacytoid DCs, which were shown to produce IL-10 and express high levels of ICOSL, as well as having the ability to induce Treg generation (121–123). A deficiency of ICOS in CD4^+^ T cells or pretreatment of DCs with anti-ICOSL mAb both abolished the ability of DCs to induce T cell tolerance *ex vivo* and in an adoptive transfer model (36). Furthermore, several studies regarding Fms-like tyrosine kinase 3 ligand (Flt3-L), which can reverse allergen-induced mouse models of asthma, demonstrated that Flt3-L not only increases the number of CD4^+^CD25^+^FOXP3^+^IL-10^+^ICOS^+^ Tregs but also recruits more CD11c^high^CD11b^low^ DCs to the lung of OVA-sensitized mice, both of which worked synergistically to attenuate AHR through the reinforced interaction between ICOS and ICOSL (124, 125).

Apart from the interaction of ICOS and ICOSL between DCs and Tregs, the interaction between Tregs and ILC2 is also important in the suppression of allergens. As is reported, group 2 innate lymphoid cells (ILC2s), which resemble Th2 cells, are capable of producing copious amounts of the type 2 cytokines IL-5 and IL-13 and are responsible for the development of allergic respiratory inflammation (126). The co-expression of ICOS and ICOSL on ILC2s promotes the homeostasis and function of these cells in a *cis* and *trans* formation, exaggerating the inflammatory responses (127, 128). In contrast, ICOS expressed on Tregs could occupy ICOSL on ILC2s, restricting the *cis* communication among ILC2s and leading to decreased cytokine production by ILC2s (127, 128). With the assistance of the suppressive cytokines TGF-β and IL-10, Tregs were shown to powerfully attenuate airway hyperreactivity induced by ILC2 in an ICOS:ICOSL cell-to-cell contact manner (127, 128).

Moreover, emerging evidence has shown that IL-35 produced by ICOS^+^ Tregs can suppress IL-17-dependent airway hyperresponsiveness, which is another synergetic mechanism contributing to allergic asthma (129). Overall, ICOS^+^ Tregs work with multiple types of immune cells to establish immune tolerance to asthma, which demonstrates their utility as a therapeutic or in the strategies for the prevention of allergic airway diseases. However, it should also be noticed that divergent outcomes could be caused by intervention of the ICOS pathway at different time points in the course of asthma. The blockade of ICOS co-stimulation was shown to attenuate Th2-mediated airway inflammation in an allergic mouse model after mice were sensitized and challenged to an allergen, while they showed symptoms comparable to allergic mice, such as aggravated eosinophil infiltration, Th2 cytokine secretion and mucus hypersecretion, if these mice received anti-ICOSL mAb in the induction stage of immune tolerance (39, 130). Therefore, both the intervention time and overall understanding of a particular disease are necessary concerns if applying drugs or other means to interfere with ICOS signaling for treatment.

### ICOS^+^ Tregs and Other Autoimmune Diseases

In addition to the three diseases mentioned above, there are other diseases with high ICOS expression on Tregs, including systemic lupus erythematosus (SLE), rheumatoid arthritis (RA), sarcoidosis (131–133). The increased frequency of ICOS^+^ Tregs has been shown to have practical significance in these diseases. For example, the elevated frequency of ICOS^+^ Tregs was observed to show a positive correlation with SLE disease activity index scores and the serum antibody titer of anti-dsDNA, although the authors suggested that these ICOS^+^ Tregs might be precursor inflammatory cells (131). In addition, it was also demonstrated to serve as a predictor of responses to treatment, as a significantly larger proportion of ICOS^+^ Tregs and skewed type 2 responses were observed in MTX-non-responsive RA patients (132). Moreover, Sakthivel and colleagues reported high expression levels of ICOS in lung Tregs of pulmonary sarcoidosis patients, and it was particularly high in patients with Lofgren’s syndrome (LS) compared with NLS, thus associating the degree of ICOS expression on Tregs with prognosis of sarcoidosis (133). In summary, the abnormal frequency of ICOS^+^ Tregs could be a potential biomarker in the assessment of prognosis and the effectiveness of specific treatment regimens.

### ICOS^+^ Tregs and Malignant Tumors

In contrast to most autoimmune diseases or allergic diseases, Tregs infiltrated in tumor tissues consistently impede effector T cell-mediated anti-tumor responses, inhibiting the treatment of tumors. ICOS^+^ Tregs, which were suggested to have a potent suppressive ability compared with their ICOS^–^ counterpart, therefore, play a dominant role in the process of immune escape. Recently, with a growing numbers of studies focusing on the immunophenotype of TILs, an increased percentage of ICOS^+^ Tregs have been observed in more and more types of tumor tissues, including melanoma, (134) head and neck squamous cell cancers, (135) gastric cancers, (136) breast cancer, (137) ovarian cancer, (138) clear cell renal cell carcinoma, (139) and acute myeloid leukemia (AML) (140). Furthermore, the elevated proportion of ICOS^+^ Tregs was shown to be associated with a bad outcome in most cases, and it was indicated as a better predictor of prognosis than the percentage of total Tregs under some circumstances. For instance, Nagase et al. observed an increased expression of ICOS in CD4^+^FOXP3^+^ TILs with the increase in the stage of gastric cancers, and the elevated expression of ICOS in CD4^+^FOXP3^+^ TILs was negatively correlated with relapse-free survival time (136). AML patients with high frequency of ICOS^+^ Tregs have an evidently shorter overall survival and disease-free survival relative to those of low ICOS^+^ Treg group (140). Moreover, an enhanced expansion of ICOS^+^ Tregs in patients with melanoma after the first cycle of high-dose IL-2 therapy was identified to be associated with unfavorable prognosis (141). In addition, the expression of ICOSL in malignant tumor cells or tumor-associated pDCs was confirmed to be a good booster for the accumulation of ICOS^+^ Tregs in some tumor tissues (137, 140, 142). Thus, the interaction between ICOS and ICOSL is a central mechanism in tumor immune evasion, although there are still other cancer types with higher levels of ICOS expression on activated effector TILs other than Tregs, in which ICOS expression was correlated with improved survival, such as in colorectal cancer (143) and lung adenocarcinoma (144).

## ICOS-Associated Therapeutic Applications in Immune Diseases

Currently, various monoclonal antibodies have been developed to intervene the overactive ICOS-ICOSL interaction. At present, there are six anti-ICOS monoclonal antibodies [JTX-2011 (NCT04319224), GSK3359609 (NCT04128696), vopratelimab (NCT04319224), BMS-986226 (NCT03251924), MEDI-570 (NCT02520791), and KY1044 (NCT03829501)] and three anti-ICOSL mAbs [AMG-557 (NCT01683695), AMG570 (NCT04058028), and ALPN-101 (NCT04227938)] on the market or in clinical trials. Treatment with anti-ICOS agonistic mAb or anti-ICOS antagonistic mAb to treat cancers by enhancing the function of effector T cells and/or depleting ICOS^hi^ Tregs alone or in combination with other monoclonal antibodies such as nivolumab and ipilimumab has achieved great success and has been a hot spot in cancer immunotherapy (145). For example, KY1044 can preferentially deplete ICOS^high^ Tregs via antibody dependent cell-mediated cytotoxicity (ADCC) and stimulate ICOS^+^ T effector cells to exhibit anti-tumor responses (146). Furthermore, some bispecific antibodies are currently being developed, including XmAb23104 (NCT03752398) and KY1055 (146), which simultaneously target ICOS/PD-1 or ICOS/PD-L1, respectively, potentially delivering a stronger anti-tumor response.

Except for these innovating monoclonal antibodies, there are other therapeutic schedules available depending on the ICOS-ICOSL pathway. The CAR with the ICOS-4-1BB fusing protein increased CAR-T cell persistence *in vivo* and anti-tumor efficacy (147). Additionally, a technology using a ^89^Zr-DFO-ICOS mAb as a probe for PET imaging was developed as a non-invasive strategy to monitor or allow for the early prediction of therapeutic responses by quantifying the number of ICOS^+^ activated T cells in a Lewis lung cancer model (148). In other words, there is great potentiality for the use of ICOS as a therapeutic target in the treatment of tumors.

For autoimmune diseases, the use of ICOS as a therapeutic target is still being explored. Despite the application of drugs to increase the proportion of ICOS^+^ Tregs or the adoptive transfer of Tregs having been shown to be therapeutic in mouse models, these treatments still have a long way to go to be put into clinical use, partly because of the technical difficulty and high cost of adoptive transfer therapy. Anti-ICOS/ICOSL mAb treatment may be a potential therapeutic option for some refractory or severe autoimmune diseases. At present, there are some phase I/II clinical trials of anti-ICOSL monoclonal antibody drugs to treat autoimmune diseases, such as on SLE, RA, psoriasis, and primary Sjögren’s syndrome. For example, AMG557 has been used for SLE treatment and shows safety and potential efficacy for this disease (149, 150). Yet a great deal of research needs to be performed before these monoclonal antibody drugs are applied to the clinic.

Additionally, it should be noted that both ICOS monoclonal antibody treatment and other therapeutic schedules act on the total ICOS signaling, rather than Treg-specific ICOS signaling. But the benefits of ICOS immunotherapy can be partly gained either by limiting ICOS^+^ Treg expansion and function in cancer diseases or by enhancing Treg immunoregulatory capacity in autoimmune diseases, although they could also be obtained by regulating effector T cells or Tfh cells, which depends on different drug mechanisms and context conditions. Different monoclonal antibodies can function through different mechanisms, affecting either effector T cells or Tregs, and diverse disease conditions should also be taken into consideration in the selection of monoclonal antibodies.

## Conclusion

The ICOS signaling pathway is widely involved in immune responses, indicating an activated state for immune cells in general. Despite exerting pro-inflammatory effect in effector T cells, ICOS signaling is also highly involved in anti-inflammatory responses, and this involvement is primarily mediated by Tregs. The results of many recent studies have suggested that ICOS^+^ Tregs are an activated subset with strong inhibitory ability that can prevent the onset or restrain the progression of most autoimmune and allergic diseases, while they can also contribute to the immunosuppression of tumors. Sufficient evidence has demonstrated that ICOS^+^ Tregs can serve as a biomarker for clinical outcome and be used in testing therapeutic responses, not only in autoimmune diseases but also in tumors. However, due to the complex and comprehensive effects of ICOS to immune cells, which are not limited to Tregs, a thorough understanding of the essential role of ICOS in Tregs and its complicated function to the overall immune system before treatment would be conducive to the formulation of rational strategies to manipulate ICOS signals, such as in the selection of appropriate anti-ICOS/ICOSL monoclonal antibody drugs.

## Author Contributions

D-YL wrote the manuscript. X-ZX conceived the idea and revised the manuscript. All authors contributed to the article and approved the submitted version.

## Conflict of Interest

The authors declare that the research was conducted in the absence of any commercial or financial relationships that could be construed as a potential conflict of interest.
