# Corrigendum: ICOS+ Tregs: A Functional Subset of Tregs in Immune Diseases

**DOI:** 10.3389/fimmu.2021.701515

**Published:** 2021-05-12

**Authors:** Dan-Yang Li, Xian-Zhi Xiong

**Affiliations:** Department of Respiratory and Critical Care Medicine, NHC Key Laboratory of Pulmonary Diseases, Union Hospital, Tongji Medical College, Huazhong University of Science and Technology, Wuhan, China

**Keywords:** ICOS, Treg cells, autoimmune disease, neoplasm, immunotherapy

In the published article, there was an error in the affiliations. Instead of “^1^ *Key Laboratory of Respiratory Diseases, National Ministry of Health of the People’s Republic of China, National Clinical Research Center for Respiratory Disease, Wuhan, China*, ^2^ *Department of Respiratory and Critical Care Medicine, Union Hospital, Tongji Medical College, Huazhong University of Science and Technology, Wuhan, China”, it should be “Department of Respiratory and Critical Care Medicine, NHC Key Laboratory of Pulmonary Diseases, Union Hospital, Tongji Medical College, Huazhong University of Science and Technology, Wuhan, China”*.

The authors apologize for this error and state that this does not change the scientific conclusions of the article in any way. The original article has been updated.

